# Supplementary material for: Pb-Free Cs3Bi2I9 Perovskite as a Visible-Light-Active Photocatalyst for Organic Pollutant Degradation
Source: Nanomaterials (Basel). 2020 Apr 16;10(4):763. doi: 10.3390/nano10040763 (PMC7221557; doi:10.3390/nano10040763)
Supplement: Supplementary file 1 [file nanomaterials-10-00763-s001.pdf]

nanomaterials

Article

SUPPORTING INFORMATIONS

Pb-Free $Cs_3Bi_2I_9$ Perovskite as a Visible-Light-Active Photocatalyst for Organic Pollutant Degradation

Bianca-Maria Bresolin [1,*], Carsten Günnemann [2], Detlef W. Bahnemann [2,3,4] and Mika Sillanpää [5,6,7]

[1] Department of Separation Sience, School of Engineering Science, Lappeenranta University of Technology, Sammonkatu 12, 50130 Mikkeli, Finland
[2] Institute of Technical Chemistry, Leibniz University Hannover, Callinstraße 3, D-30167 Hannover, Germany; guennemann@iftc.uni-hannover.de (C.G.); Bahnemann@iftc.uni-hannover.de (D.W.B.)
[3] Laboratory of Nano- and Quantum-Engineering (LNQE), Gottfried Wilhelm Leibniz University Hannover, Schneiderberg 39, D-30167 Hannover, Germany
[4] Laboratory ''Photoactive Nanocomposite Materials'', Saint-Petersburg State University, Ulyanovskaya str. 1, Peterhof, 198504 Saint-Petersburg, Russia
[5] Institute of Research and Development, Duy Tan University, De Nang 550000, Vietnam; mikaesillanpaa@gmail.com
[6] Faculty of Environmental and Chemical Engineering, Duy Tan University, De Nang 550000, Vietnam
[7] School of Civil Engineering and Surveying, Faculty of Health, Engineering and Science, University of Southern Queensland, West Street, Toowoomba 4350, QLD, Australia
* Correspondence: biancabresolin@yahoo.it or Bianca.Maria.Bresolin@lut.fi

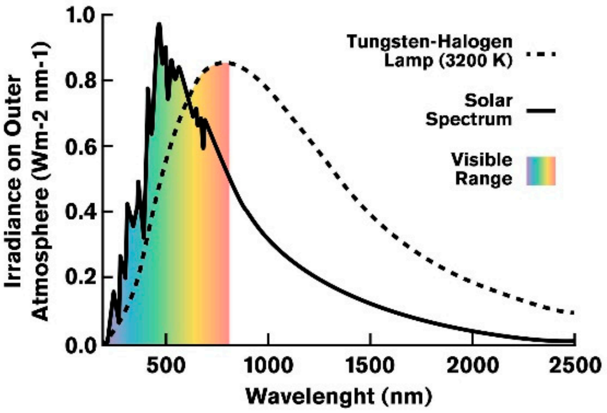

Figure S1. Irradiance spectrum of a Halogen lamp (3200K) compared to the solar irradiance on a surface per unit wavelength with emphasis on visible light range.

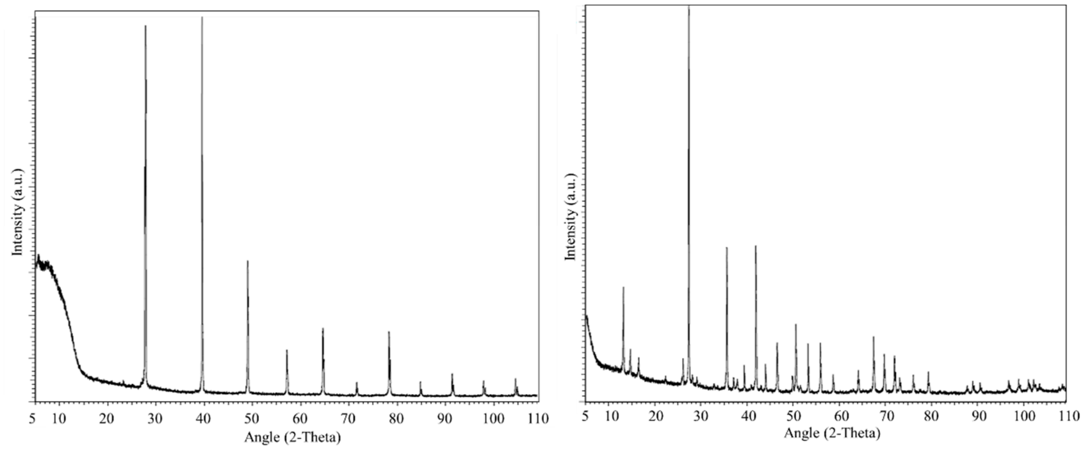

Figure S2. XRD patterns of CsI and BiI3.

© 2020 by the authors. Submitted for possible open access publication under the terms and conditions of the Creative Commons Attribution (CC BY) license (http://creativecommons.org/licenses/by/4.0/).
